# The ecological discourse analysis of news discourse based on deep learning from the perspective of ecological philosophy

**DOI:** 10.1371/journal.pone.0280190

**Published:** 2023-01-25

**Authors:** Biyun Zhang, Shanti Chandran Sandaran, Jing Feng

**Affiliations:** 1 Language Academy, Faculty of Social Sciences and Humanities, Universiti Teknologi Malaysia, Johor Bahru, Malaysia; 2 School of Foreign Languages, Yan’an University, Yan’an, Shaanxi, China; Hanyang University, REPUBLIC OF KOREA

## Abstract

Recently, ecological damage and environmental pollution have become increasingly serious. Experts in various fields have started to study related issues from diverse points of view. To prevent the accelerated deterioration of the ecological environment, ecolinguistics emerged. Eco-critical discourse analysis is one of the important parts of ecolinguistics research, that is, it is a critical discourse analysis of the use of language from the perspective of the language’s ecological environment. Firstly, an ecological tone and modality system are constructed from an ecological perspective. Under the guidance of the ecological philosophy of "equality, harmony, and symbiosis", this study conducts an ecological discourse analysis on the Sino-US trade friction reports, aiming to present the similarities and differences between the two newspapers’ trade friction discourses and to reveal the ecological significance of international ecological factors in the discourse. Secondly, this method establishes a vector expression of abstract words based on emotion dictionary resources and introduces emotion polarity and part-of-speech features of words. Then the word vector is formed into the text feature matrix, which is used as the input of the Convolutional Neural Network (CNN) model, and the Back Propagation algorithm is adopted to train the model. Finally, in the light of the trained CNN model, the unlabeled news is predicted, and the experimental results are analyzed. The results reveal that during the training process of Chinese and English datasets, the accuracy of the training set can reach nearly 100%, and the loss rate can be reduced to 0. On the test set, the classification accuracy of Chinese text can reach 83%, while that of English text can reach 90%, and the experimental results are ideal. This study provides an explanatory approach for ecological discourse analysis on the news reports of Sino-US trade frictions and has certain guiding significance for the comparative research on political news reports under different ideologies between China and the United States.

## 1. Introduction

Global climate change, as one of the most severe "ecological crises" facing mankind in the 21st century, deserves enough attention from the international community. In the context of advocating a community of shared future for mankind, it is the social responsibility and historical mission of linguists to discuss climate change from the perspective of ecolinguistics. Therefore, the research on the ecological discourse of climate change has both theoretical and practical significance. As the primary source for the public to obtain information, news discourse can transmit certain attitudes, intentions, and emotions, thus affecting their cognition and behavior. Thereupon, news discourse on climate change plays an important role in guiding media opinion, shaping public perception, and facilitating the formulation of climate policies [1, 2].

The rapid progress of Internet-related technologies has brought quiet changes to people’s daily life. It has become a part of daily life for users to express their views on social networks through microblogs and other we-media, and to evaluate online products on e-commerce platforms. It has become an important research issue in the field of artificial intelligence how to analyze such texts by using machine learning (ML) and natural language processing technology to obtain the viewpoint orientation and emotional polarity in them. Traditional sentiment analysis techniques can be roughly divided into rule-based approaches and statistics-based methods [3, 4]. The rule-based approach is mainly from the perspective of linguistics, using the knowledge of language experts to compile dictionaries and templates to analyze the emotional tendencies in the text. The statistics-based method starts from the point of ML, uses the manually labeled training corpus for feature extraction and statistical model construction, and automatically realizes the judgment of emotional polarity. In the development of text sentiment analysis for more than ten years, the two kinds of methods have infiltrated each other, making sentiment analysis technology move towards a higher level. In the process, researchers have amassed a wealth of resources, tools, algorithms, and models. The method based on the Deep Neural Network (DNN) is an automatic sentiment analysis method. Due to the large scale of the model parameters, the construction and search of the feature space and the establishment of the model are more refined, and the performance also reflects the advantages compared with the previous methods. But this kind of method neglects the effective use of the existing accumulated emotional resources, including the existing rules, the existing emotional dictionary, the knowledge base, and so on. How to integrate the DNN and existing effective computing resources is a problem to be solved [5, 6].

The innovation of this study is that it proposes an algorithm based on the Convolutional Neural Network (CNN) to mine the sequence features of text emotional tendencies. By using the inherent features of the words themselves, the text is mapped into a low-dimensional abstract feature matrix, which reduces the complexity of the CNN model and speeds up the training of the model on the basis of ensuring the recognition performance of the emotional orientation of the text. In addition, the proposed algorithm can mine the sequence features from the text that represent the emotional orientation, which can provide other models with useful feature information for emotion classification.

## 2. Related work

Previous studies of news discourse on climate change mainly focus on two academic approaches. One is to carry out research along the path of journalism and communication, focusing on guiding the media opinion environment, shaping the national image, and disseminating media information. The other is along the path of linguistics, such as the study of language features from critical discourse analysis and ecological discourse analysis, thereby exploring the role of language in mapping climate reality, building climate discourse power, and deepening the process of climate governance. The ecological discourse of news discourse of climate change is mainly studied from the aspects of evaluation theory, approach theory, and framework & metaphor theory. Zhang (2020) [7] pointed out that quantitative discourse analysis tools had become a growing popular method to operate various corresponding types. However, their applications are often simplistic and superficial and do not take full advantage of the discourse analysis resources available in the digital age. They discussed the potential for discourse analysis of a more sophisticated, advanced text analysis tool that is already frequently used in other similar methods, especially topic modeling. Chen and Wang (2019) [8] used critical discourse analysis and translation theory to analyze the discourse strategies in news reporting, to discover the ideology hidden in the reporting discourse beyond the surface language symbols. To this end, they selected 674 discourses from the Cable News Network’s report on the "Occupy Central" movement in Hong Kong from September 26, 2014, to October 26, 2014, as the research object. The study argues that opponents and supporters of the movement represent centripetal and centrifugal forces, respectively. The reporting discourse strategy seems objective and fair, but by setting various character frames for different quoted speakers, selectively using reporting verbs, and using the pragmatic functions of different reporting modes to convey ideology subtly. Chang-Chen and Zhang (2018) [9] used ecological discourse analysis to analyze the ecotourism texts of Jiuzhaigou scenic spots from the aspects of transitivity and attitude resources. They found that its purpose is to render a good ecological environment of ecotourism scenic spots, to create a harmonious, equal, and friendly atmosphere between man and nature, highlighting the attractiveness of eco-tourism scenic spots. Wang (2018) [10] compared the news reports on climate change between China and the US from the perspective of eco-critical discourse analysis, involving news headlines, word choices, and topics, and discussed the differences in their respective positions and ideologies in the new reports of climate change between the two countries, to ultimately improve people’s ability to compare and analyze discourse and awareness of language ecology protection. Wei and Geng (2018) [11] conducted an ecological analysis of a children’s milk advertising discourse. Advertisers construct beneficial discourses and advocate the ideology of healthy consumption and ecological harmony. The findings suggest that ecological analysis of advertising discourse can reveal economic behavior and influence the public’s consumption behavior. Chang and Cong (2018) [12] conducted a comparative analysis of English and Chinese environmental protection advertisements based on the sense of harmonious ecological place and the theory of transitive systems. The purpose is to reveal the similarities and differences in the expression of ecological attributes between English and Chinese advertisements and to provide suggestions for advertisement production. The results indicate that English advertisements mainly use psychological processes to express their ecological attributes, emphasizing the establishment of an emotional connection between man and nature, while Chinese advertisements mainly use action processes to express ecological attributes, reflecting the connotation of man to nature. Huang et al. (2022) [13] pointed out that the competitive group optimizer is an effective variant of the Particle Swarm Optimization (PSO) algorithm, which has been widely applied to deal with various practical large-scale optimization problems. They introduced a new multi-stage co-evolution technique and proposed a new three-phase co-evolution method to improve the convergence and search capabilities of the PSO algorithm. Song et al. (2023) [14] designed a differential evolution (DE) algorithm with a dynamic mixing mechanism of quantum evolutionary algorithm (QEA) and genetic algorithm (GA) to schedule trains and reduce delays. When the algorithm is improved, according to the co-evolutionary performance, the quantum variable decomposition strategy was designed by using qubit string adaptive variable decomposition. On the basis of making full use of the evolutionary information, an incremental mutation algorithm is proposed to improve the convergence speed of the algorithm. In order to improve the accuracy and generalization ability of hyperspectral image classification, Chen et al. (2021) [15] proposed a feature extraction method for hyperspectral images combining principal component analysis (PCA) and the local binary pattern (LBP). First, PCA is used to reduce the spectral features of hyperspectral images. Then, the Grey Wolf Optimizer (GWO) with global search ability is adopted to optimize the parameters of the nuclear extreme learning machine, the optimized nuclear extreme learning machine model is implemented. Li et al. (2022) [16] argued that parametric time-frequency analysis can effectively improve the time-frequency energy aggregation of non-stationary signals and the disturbance resistance of cross-term interference, but there is energy diffusion near the real instantaneous frequency. The improved multi-synchronous compression transform can improve the time-frequency energy aggregation, but there are still some defects in processing strong FM and AM signals under noise interference. Thereupon, to make use of their advantages and overcome their disadvantages, a new parameterized multi-synchronization compression transform method based on weighted least squares is proposed. The experimental results testify that this method can effectively use the analog signal and the actual fault signal to process the non-stationary signal with instantaneous frequency change. After a literature review, it is found that the existing studies have the following four deficiencies. First, it focuses on qualitative analysis, with less empirical quantitative analysis; Second, it focuses on the description of language features, and seldom involves the discussion of social and cultural factors. Third, it focuses on the perspective of critical discourse analysis, rather than the perspective of ecological discourse analysis; Fourth, it focuses on the media reports of a single country and rarely involves the comparative study of Chinese and foreign news media.

At present, feature extraction methods based on artificial rules and dictionary resources often rely on a specific domain or specific corpus. Furthermore, the text feature dimension increases linearly with the increase of artificial rules and dictionary resources, which not only increases the training cost of the model but also reduces the generalization ability of the model. To realize the effective mining and expression of the emotional features of text, a CNN method combining the emotional features of words is proposed. This method first establishes an abstract word vector expression method based on emotion dictionary resources and introduces emotional polarity and speech characteristics of words. Then word vectors are formed into a text feature matrix, which is used as the input of the CNN model, and the Back Propagation (BP) algorithm is employed to train the model.

## 3. Ecolinguistics and ecological discourse analysis

### 3.1 Ecological philosophy from the perspective of climate change theory

The concept of ecological philosophy refers to "an idea to examine the relationship between living things, inanimate things, and the natural environment, including human beings". In order to objectively evaluate the ecological value orientation contained in the discourse, the analyst of ecological discourse needs to ensure a prerequisite [17–20]. That is, on the basis of absorbing ecological philosophy or ethical thoughts related to the relationship between man and nature, integrating and innovating these thoughts in combination with the actual situation and specific problems, establishing a scientific and unified ecological philosophy view suitable for a specific context, and taking it as a yardstick to measure the ecological orientation of discourse. In the ecosystem, humans, animals, plants, microorganisms, inanimate organisms, and other ecological factors will have an effect on the climate. In turn, they are also affected by climate, and the interaction between these ecological factors is a cyclic process. The ecological environment is the basic condition of human survival, and also the precondition of sustainable development. The situation of climate change is becoming more and more serious. Any environmental policy formulation should adhere to the principle of "ecological priority", pursue "green development", practice green ideas, and take concrete actions to protect the climate and environment. The relationship between man and nature is a "harmonious coexistence". Nature is the source of human life, and human beings should respect, comply with and protect nature. In the face of the climate crisis, the international community needs to jointly build a "community of life on Earth". Ecological discourse analysis focuses on the ecological attributes of discourse and needs the guidance of universal ecological philosophy. Therefore, this study will follow the ecological philosophy of "green development, harmonious coexistence" from the point of climate change theory, and judge the ecological attributes contained in the news discourse, so as to effectively screen the beneficial, ambiguous, and destructive discourse [21–23].

### 3.2 Ecological discourse analysis framework in news discourse

As a major research paradigm of ecolinguistics, ecological discourse analysis extends the research object of discourse analysis to the whole ecosystem covering the social system. It aims to clarify the ecological factors that are not conducive to the harmonious development of man and nature in the discourse and urge humans to reflect on and correct the ecological crisis, thereby enhancing the awareness of environmental protection and helping the balance of the ecological system. Following the method of "description-explication-explanation", the study conducts an in-depth analysis of climate change news discourses. Specifically, the description approach refers to the analysis of the language features in news discourses on climate change, the explication approach stands for the analysis of the process of news discourses’ production, and the explanation approach refers to the rational exploration of abstract social and cultural factors. These three dimensions have intrinsic and self-consistent logical correlation, and point-to-surface linkage analysis will be more conducive to the systematic grasp of ecological discourse [24, 25].

**Fig 1:**
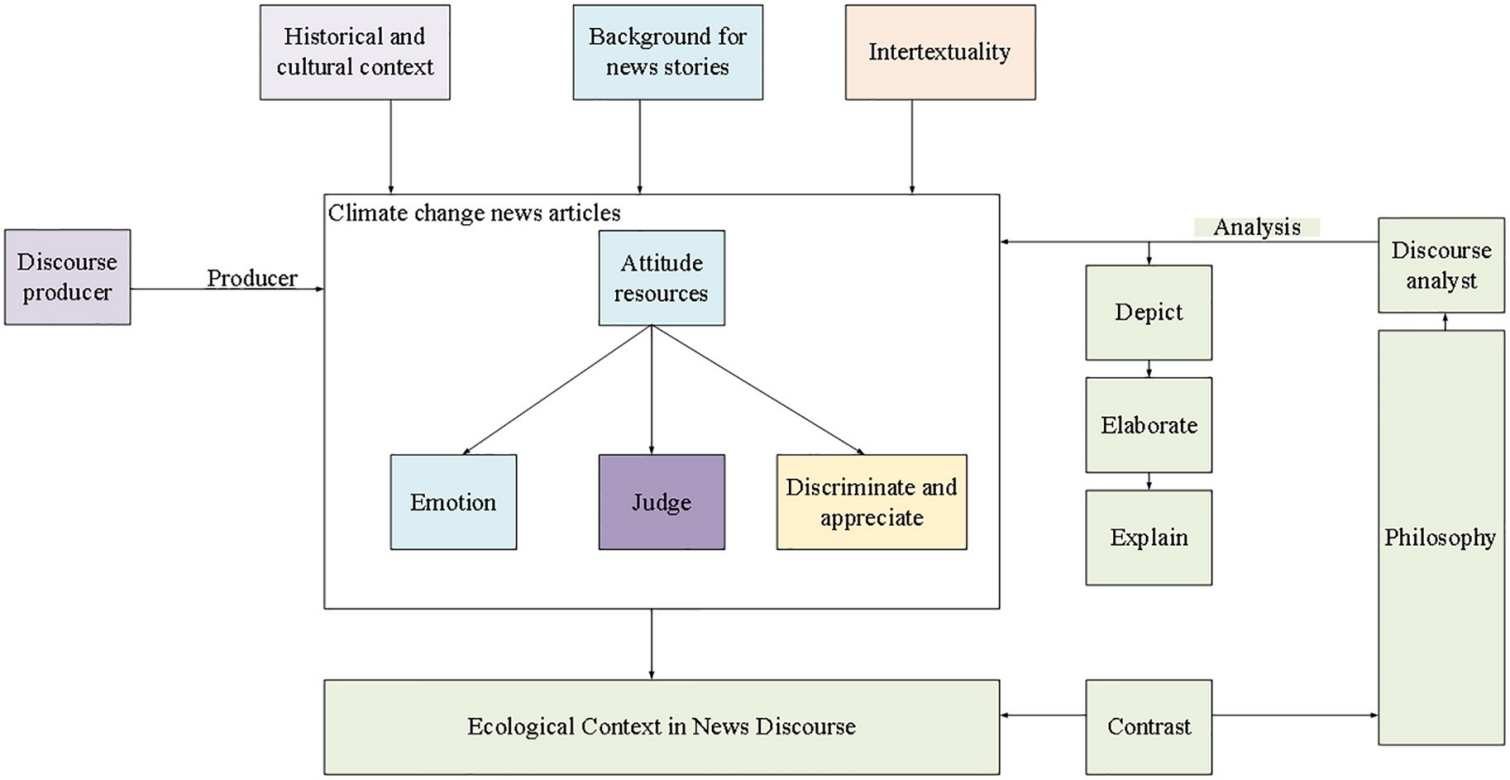
Ecological discourse analysis framework of attitude resources in news discourse.

The ultimate goal of this study is to conduct a detailed and in-depth ecological discourse analysis of the attitude resources in the news discourses on climate change, so as to realize the independent judgment of the ecological information conveyed by the news discourses on climate change. In other words, discourse analysts compare the ecological concept presented by attitude resources with the ecological philosophy under the climate change theory to determine whether the two are compatible, thus identifying the ecological value orientation in the news discourse on climate change. The consonant is beneficial discourse [26, 27], the neutral is an ambiguous discourse, and the contravention is destructive discourse. Therefore, this study constructs an ecological discourse analysis framework for attitude resources in the news of discourses climate change. Fig 1 presents the ecological discourse analysis framework of attitude resources in news discourse.

### 3.3 Extraction model of the emotional sequence features of words based on CNN

ML is an algorithm that analyzes data rules by means of algorithms and predicts results by using rules. It is divided into supervised learning, unsupervised learning, and reinforcement learning. Deep learning (DL) is the extension of the neural network algorithm in ML, and it is the second stage of ML, where depth refers to the number of layers of the neural network [28, 29]. With the development of ML, the ability of ML to automatically learn data implied high-level features will be gradually improved with the improvement of the model and the expansion of training data, which leads to the development of DL. The neural network is an algorithm in DL, and it is also the carrier of DL [30–33]. It contains an input layer, a neuron layer, and an output layer. The earliest single-layer neural network is also called perceptron. Structurally, it is composed of the input layer, hidden layer, and output layer, and the weight between each layer is connected.

In order to realize the effective mining and expression of the emotional features of text, this study proposes a CNN method combining the emotional features of words. Firstly, this method establishes an abstract word vector expression method based on emotion dictionary resources, through which emotional polarity and speech characteristics of words are introduced. Secondly, word vectors are formed into a text feature matrix, which is used as the input of the CNN model, and the BP algorithm is adopted to train the model. Finally, the sequence features generated by the model are extracted and represented as the emotional features of the input text and added to the Support Vector Machine (SVM) classifier to achieve the emotional polarity of the text [34–36].

Firstly, a method of word vector construction using dictionary resources is proposed. Different from the representation of real vectors such as word embedding (word2vec), this study uses the attribute characteristics of the word itself to construct the word vector.

Each word is mapped to a *k*-dimensional 0, 1 vector space, that is, *x* ∈ R^*k*^, where *k* refers to the number of features the word itself has, and the value of each dimension is represented by 0 or 1; 0 denotes that it does not have this feature, and 1 expresses that it has this feature. For a given sentence, it contains *n* words *x*_*i*_. 1 ⩽ *i* ⩽ *n*, forming a characteristic matrix of *n* × *k*. Here, the phrase fragments composed of the *i*th to *j*th words in the sentence are recorded as *X*_[*i*:*j*]_. Similarly, sentences containing *n* words are written as *X*_[1:*n*]_.

### 3.4 CNN model based on word vector

**Fig 2:**
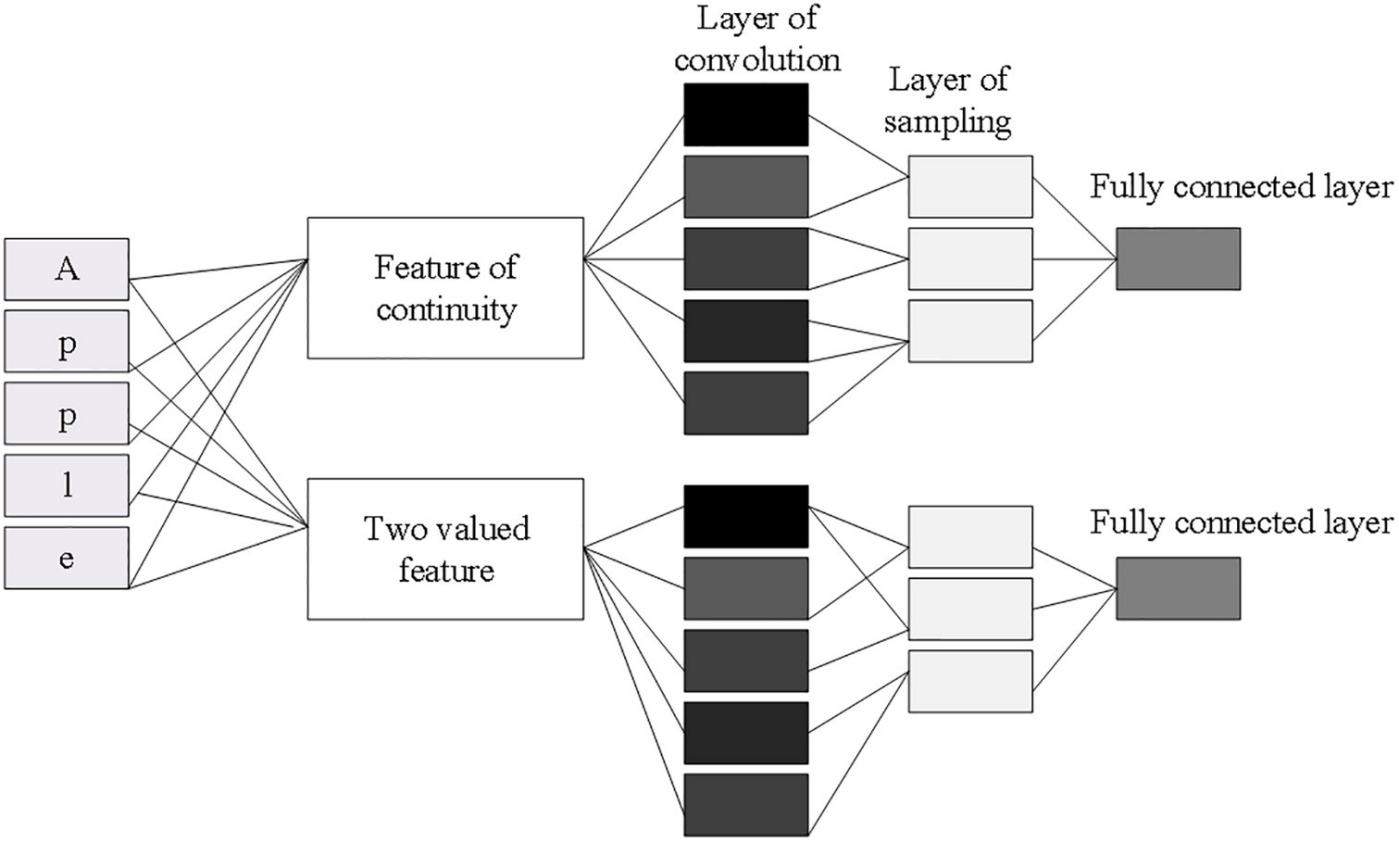
The constructed neural network model.

CNN is a feedforward neural network, which is composed of a convolutional layer and a subsampled layer. The output of each layer is the input of the next layer. As the feature extraction layer, the convolutional layer extracts local features through the filter, generates feature graphs through the operation of the convolution kernel function, and outputs them to the subsampled layer. The subsampled layer is a feature mapping layer, which samples the feature map generated by the convolutional layer and outputs local optimal features. Fig 2 plots the CNN model adopted in this study.

A filter of size *h* × *k* is used to perform convolution operation to the input characteristic matrix, and the mathematical relationship is shown in Eq (1).

$$c_{i}=f\left(w\cdot X_{i:i+h-1}+b\right)$$ (1)

*c*_*i*_ represents the ith eigenvalue in the characteristic graph; *f*(∙) refers to the convolution kernel function, *w* ∈ *R*^*hk*^ is the filter; *h* stands for the sliding window size; *b* means the offset value; *X*_*i*:*i*+*h*−1_ expresses the local characteristic matrix consisting of *ith* rows to *i* + *h* − 1 row. Therefore, the mathematical representation of characteristic diagram *C* is indicated in Eq (2):

$$C=\left[c_{1},c_{2},c_{3},\cdots c_{n-h+1}\right]$$ (2)

The subsampled layer adopts the max over pooling method proposed by Golubert for feature sampling, and the eigenvalue obtained is $\hat{c}$. Its mathematical expression is illustrated in Eq (3):

$$\hat{c}=max\left\{C\right\}$$ (3)

The convolutional layer and the subsampled layer constitute the feature extraction layer of the model. The model consists of several different types of feature extraction layers (*h* take different values) and the composition of each type of feature extraction of each *m* a layer. Thus, the mathematical expression of feature vector *V* of the Fully Connected Layer (FCL) is implied in Eq (4):

$$V=\left[{\hat{c}}_{1,h_{1}},\cdots ,{\hat{c}}_{m,h_{1}}\cdots ,{\hat{c}}_{1,h_{j}},\cdots ,{\hat{c}}_{l,h_{j}},\cdots \right]$$ (4)

${\hat{c}}_{l,h_{j}}$ represents the *l*th eigenvalue generated by the jth type of filter. It is expected that based on the proposed word feature representation, word sequence features related to positive and negative emotion labels can be further extracted for the final emotion classification through this network structure.

The feature vector output from the subsampled layer is used as the input of the FCL, and then Softmax is used to output the classification results. According to the actual classification label of the training data, BP algorithm is employed to carry out gradient update of the model parameters, as expressed in Eq (5):

$$\left.P_{(y}|V,W_{s},b_{s}\right)={\text{softmax}}_{y}\left(W_{s}\cdot V+b_{s}\right)$$ (5)

*y* ∈ {+1, −1}; *W*_*s*_ ∈ R^|*v*|^; *b*_*s*_ is the offset term.

Finally, the trained model is adopted to transform the text feature matrix into feature vector *V*, and the classifier is used for model training and classification.

### 3.5 Word sequence feature fusion method

After feature extraction of the text feature matrix, the proposed model outputs the text feature vector *V*. The feature vector *V* can be used as an additional feature of other models, providing a simple and efficient method for other models to embed text emotional orientation features. It can be assumed that $\hat{V}$ is the feature vector after adding the emotional tendency features of the text, *V*_original_ is the original feature vector of the model, so there is the relationship of Eq (6):

$$\hat{V}=V_{\text{original}\;}⊕V$$ (6)

⊕ refers to the vector splicing operation.

Under the proposed computational system, each word in the text can be abstracted, and the discrete features can be selected to represent its possible emotional polarity and other properties that may reverse or enhance the emotional polarity. On this basis of this, through the convolution operation of CNN, feature extraction based on convolution operation is carried out on the abstracted word attribute sequence. Through this operation, sequence features based on abstract attributes are obtained. For example, the rule "negative + negative polarity = positive polarity" can be obtained through convolution operation by combining sequence features and text classification labels.

### 3.6 Experimental setup and dataset description

The experiments are performed under the Windows 10 system, the used Central Processing Unit (CPU) is Inter Core i5-2450M 2.5GHz, and the size memory is 6GB. Its programming language is Python 3.0, and the development tool is Pycharm. On account of the Python programming language, news articles on climate change in China Daily, People’s Daily, The New York Times, and USA Today were selected based on comprehensive consideration of circulation and influence. Then, data cleaning, manual verification, and noise reduction were carried out on the crawled corpus, and the news discourse closest to the theme of climate change was selected. The Chinese and American climate change news corpora were built by ourselves. Taking the Trump administration’s withdrawal from the Paris Agreement in 2017 as the starting point, this study selected 279 news articles on climate change in China, with a total library of 204,049 symbols. 211 news articles on climate change in the US, with a total library of 203,828 symbols.

The adjustable parameter settings of the CNN model shown in Table 1 are adopted here. In the model training stage, Adadelta Update Rule is used to update the model parameters by stochastic gradient descent.

**Table 1:** Parameter settings of CNN.

Adjustable parameters	Value
Convolution kernel function	Rectified linear function
Size of a sliding window of filters	2, 3, 4, 5
Number of filters	100
The proportion of randomly updated parameters	0.5
Number of training iterations	50

## 4. Analysis and discussion of experimental results

### 4.1 Performance of CNN-based nonlinear excitation functions

**Fig 3:**
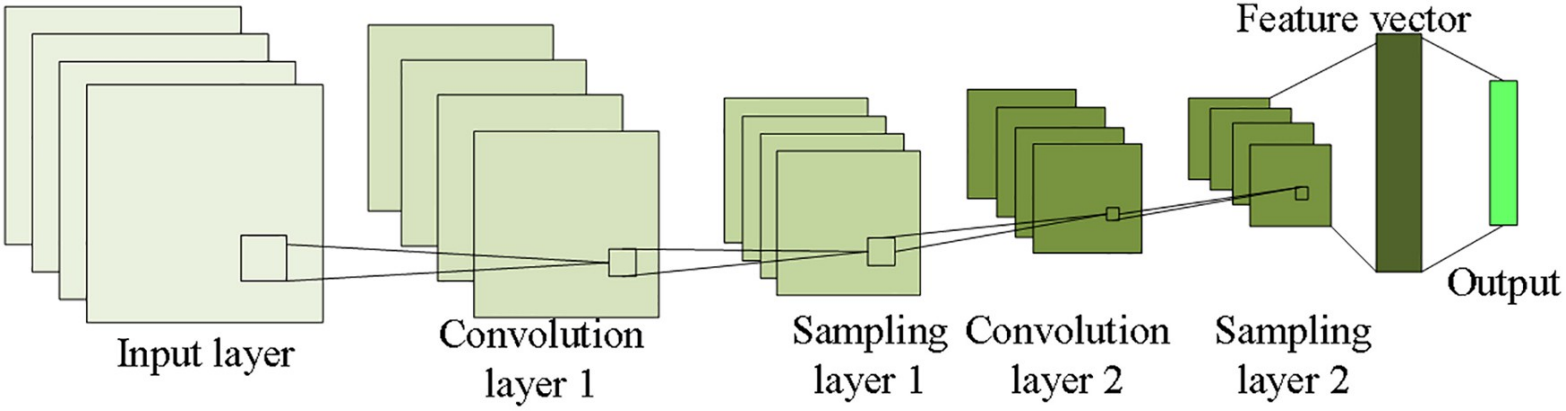
Change and distribution of nonlinear excitation function based on CNN. a. Learning rate is 0.001; b. Learning rate is 0.01.

Fig 3 denotes the distribution and changes of the saturated nonlinear and unsaturated nonlinear excitation functions by CNN.

After analyzing the curve changes in Fig 3, it means that the curves corresponding to the saturated nonlinear excitation functions Tanh and Sigmoid have the same trend, the difference lies in the difference in the value range. The output value corresponding to the curve of the ReLUs is 0 when x<0, and the ReLUs family functions correct the data in some regions of x>0. In contrast, the CNN-based P-S function is based on the training constant b, and its slope changes in real-time as the training progresses. In the end, it converges to an appropriate constant. In the composition region of x>0, the P-S function performs smooth nonlinear correction processing on the corresponding data, and its derivative is a smooth and continuously increasing function in this composition region.

According to the above analysis of the curve in the composition of the CNN structure, it can be known that the original excitation function in CNN has its own unique performance, such as the continuous differentiability of the Sigmoid, the sparse expression ability of the ReLUs, the nonlinear mapping correction ability of the Softplus, etc. The given P-S function has both sparse expression ability and nonlinear mapping correction ability. In the convolutional layer of CNN, using it has better convergence performance and can obtain the optimal solution.

### 4.2 The trend of accuracy and loss rate of the model

**Fig 4:**
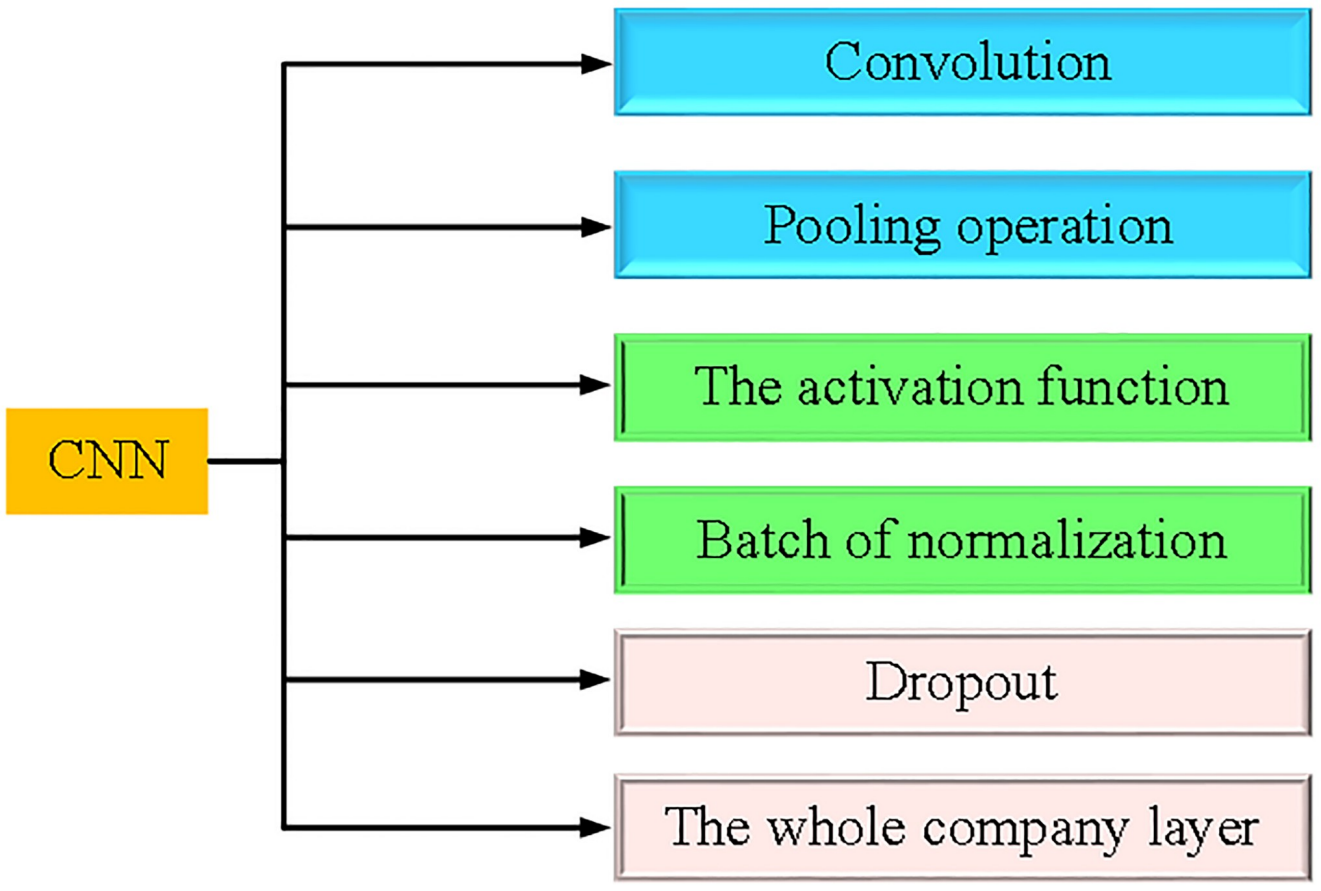
The trend of accuracy and loss rate of Chinese and English texts. a. Accuracy (Chinese); b. Loss rate (Chinese); c. Accuracy (English); d. Loss rate (English).

Fig 4 indicates the trend of the loss rate and accuracy of Chinese and English texts.

The above results testify that during the training process of the Chinese and English data sets, its accuracy can reach close to 100%, and the loss rate can be reduced to 0. On the test set, the classification accuracy of Chinese text can reach 83%, while that of English text can reach 90%, and the experimental results are ideal.

To evaluate the model, the calculation results of the model’s recall, precision, and F1 value on the test set are expressed in Table 1. To verify the effect of the used model on news text classification, it sets up a set of comparative experiments. Under the same English data set, compared with the SVM of the traditional ML model and the CNN that does not use Word2vec for word vectorization, Table 2 demonstrates the findings:

**Table 2:** Comparison of performance of the models.

Items	Text set	Precision	Recall	F1 value
CNN+Word2vec	Chinese	0.83	0.81	0.82
	English	0.91	0.88	0.88
CNN	Chinese	0.79	0.70	0.73
	English	0.77	0.72	0.76
SVM	Chinese	0.71	0.72	0.77
	English	0.68	0.66	0.64
The proposed algorithm	Chinese	0.84	0.81	0.82
	English	0.91	0.94	0.91
Word2Vec-CNN	Chinese	0.94	0.98	0.87
	English	0.92	0.99	0.90
NBSVM (Naive Bayes Support Vector Machines)	Chinese	0.88	0.87	0.86
	English	0.90	0.90	0.90

As can be seen from the experimental results in the above table, there is a certain gap in performance between the proposed model and Word2VEC-CNN based on Word2vec. However, the proposed model constructs feature inputs with low-dimensional word features. Compared with 50-dimensional or even 100-dimensional word vectors, the complexity of the model is reduced, model parameters are decreased, model training speed is accelerated, and good performance is guaranteed.

### 4.3 Experimental results of the discourse analysis

The beneficial ecological discourse database is processed through the word segmentation statistical software developed by the country, and the emotional words are sorted in descending order with the frequency of occurrence as an indicator. Table 3 presents the results of the top 10 beneficial ecological discourse-emotional words.

**Table 3:** Ranking of beneficial ecological discourse-emotional words.

Emotional words	Frequency	Frequencies	Emotional words	Frequency	Frequencies
A	0.012	8	D	0.0045	5
B	0.009	6	E	0.003	3
C	0.0075	7	F	0.0016	2

The meanings of A-F in Table 3 are forced, welcome, support, thanks, understanding, and worry.

In Table 3, the above emotional words mainly appear in the description of natural disasters destroying human well-being. By appearing at the beginning of news discourse, the purpose is to explain the ecological changes to readers, which plays a crucial role. At the same time, it guides readers to realize the importance of the ecological environment, publicizes environmental protection work, applies the power of ecological language, and stimulates the enthusiasm of readers to protect the ecological environment. Emotional words loaded with ecological change semantics can touch readers’ perceptions, so that readers can re-examine their relationship with nature and take action.

The emotional words are sorted in descending order by word segmentation statistical software, and the arrangement of neutral ecological discourse-emotional words is indicated in Table 4.

**Table 4:** Arrangement of neutral ecological discourse-emotional words.

Emotional words	Frequencies	Frequency	Emotional words	Frequencies	Frequency	Emotional words	Frequencies	Frequency
a	1	0.0038	b	1	0.0038	c	1	0.0038

In Table 4, the meanings of a-c are belief, guilt, and regret.

In the neutral ecological discourse database, the frequency of emotional words is lower than that of the beneficial ecological discourse database. There are only three words that meet the statistical conditions, namely "believe", "shame" and "regret", and the frequency is only once. Among them, the emotional word "believe" refers to more perfect data accuracy and full confidence in ecological environmental protection. From the perspective of system function, "believe" has no effect on readers’ awareness of environmental protection. The "shame" expresses a human’s reflection on the destruction of the natural environment and expresses the writer’s guilt for the destruction of the natural environment. The "regret" contains rich emotions and focuses on the writer’s disappointment in the ecological construction path, and will not affect the readers’ ecological view.

By comparing the thematic corpus collected in the news reports on climate change in China and the US, it finds the following conclusions. First, compared with the news reports on climate change in the US, it tends to take authority as the subject in China, such as the country, leaders of relevant agencies, senior officials, and others, while the U.S. choose to focus more on people. Second, differences in relevant organizations and American parties, and public opinions often appear in news reports on climate change in the US, but they rarely appear in China. Third, the topics in China’s climate change news reports will involve many countries such as the US, while the US reports rarely involve other countries.

### 4.4 The ecological orientation of attitude resource refraction in Chinese news discourse

This study evaluates the ecological orientation of attitude resources in Chinese news discourses and finds that they all present beneficial, fuzzy, and destructive ecological orientations. The analysis results of the ecological orientation of attitudinal resource refraction in news discourses of the Chinese climate change are outlined in Table 5:

**Table 5:** Ecological orientation of attitudinal resource refraction in news discourses of the Chinese climate change.

	Happiness	Satisfaction	Security	Tendency	Normalization	Capacity	Perseverance	Authenticity	Appropriateness	Reactivity	Constitutive	Value
Beneficial ecological orientation	39	65	60	33	37	212	197	67	941	89	67	1899
Fuzzy ecological orientation	0	2	2	2	2	5	4	3	17	1	0	41
Destructive ecological orientation	2	5	2	4	8	6	12	8	145	8	5	68

In Table 5, beneficial orientation outweighs fuzzy and destructive orientation in emotional resources. China tends to deliver positive voices on global climate change governance issues. Win-win international cooperation and joint efforts to deal with climate change have always been the main theme of Chinese media.

## 5. Conclusion

Ecological discourse analysis is carried out on the relevant corpus of news reports on climate change in China and the US. Specifically, from the perspective of ecolinguistics, it conducts a comparative analysis of the report title, vocabulary selection, and topic selection, to analyze the similarities and differences in the attitudes and measures taken by China and the US in dealing with the issue of global climate change. It can be found that news reports on China’s climate change objectively expressed their attitudes and views on relevant events in the title; in the choice of words in the text, more positive and affirmative words were chosen to create a responsible image for readers. In the aspect of topic selection, it is more inclined to choose authoritative organizations and personnel. In contrast, news reports on climate change in the US tend to choose the negative impact of the measures taken by their political circles on the future of the US in the headlines. The selection of text vocabulary is mostly negative and negative; the theme is mostly aimed at the US. In terms of themes, most of them are aimed at the US, and more relevant personnel such as vested interests are selected as the theme. It can be seen that it has conducted a more in-depth analysis of the news reports on climate change in China and the US. As the proposed algorithm depends on dictionary resources the quantity and quality of dictionary resources directly affect the performance of the algorithm. Therefore, how to make use of CNN’s advantages in abstract feature extraction to mine and expand dictionary resources will be the next research work.

## Supporting information

S1 File (ZIP)
