# Supplementary material for: The ecological discourse analysis of news discourse based on deep learning from the perspective of ecological philosophy
Source: PLoS One. 2023 Jan 25;18(1):e0280190. doi: 10.1371/journal.pone.0280190 (PMC9876347; doi:10.1371/journal.pone.0280190)
Supplement: S1 File — (ZIP) [file pone.0280190.s001.zip › data packet/Table 1 -Table 5.docx]

Table 1 Parameter settings of CNN

| Adjustable parameters | Value |
| --- | --- |
| Convolution kernel function | Rectified linear function |
| Size of a sliding window of filters | 2, 3, 4, 5 |
| Number of filters | 100 |
| The proportion of randomly updated parameters | 0.5 |
| Number of training iterations | 50 |

Table 2 Comparison of performance of the models

| Items | Text set | Precision | Recall | F1 value |
| --- | --- | --- | --- | --- |
| CNN+Word2vec | Chinese | 0.83 | 0.81 | 0.82 |
|  | English | 0.91 | 0.88 | 0.88 |
| CNN | Chinese | 0.79 | 0.70 | 0.73 |
|  | English | 0.77 | 0.72 | 0.76 |
| SVM | Chinese | 0.71 | 0.72 | 0.77 |
|  | English | 0.68 | 0.66 | 0.64 |
| The proposed algorithm | Chinese | 0.84 | 0.81 | 0.82 |
|  | English | 0.91 | 0.94 | 0.91 |
| Word2Vec-CNN | Chinese | 0.94 | 0.98 | 0.87 |
|  | English | 0.92 | 0.99 | 0.90 |
| NBSVM (Naive Bayes Support Vector Machines) | Chinese | 0.88 | 0.87 | 0.86 |
|  | English | 0.90 | 0.90 | 0.90 |

Table 3 Ranking of beneficial ecological discourse-emotional words

| Emotional words | Frequency | Frequencies | Emotional words | Frequency | Frequencies |
| --- | --- | --- | --- | --- | --- |
| A | 0.012 | 8 | D | 0.0045 | 5 |
| B | 0.009 | 6 | E | 0.003 | 3 |
| C | 0.0075 | 7 | F | 0.0016 | 2 |

Table 4 Arrangement of neutral ecological discourse-emotional words

| Emotional words | Frequencies | Frequency | Emotional words | Frequencies | Frequency | Emotional words | Frequencies | Frequency |
| --- | --- | --- | --- | --- | --- | --- | --- | --- |
| a | 1 | 0.0038 | b | 1 | 0.0038 | c | 1 | 0.0038 |

Table 5 Ecological orientation of attitudinal resource refraction in news discourses of the Chinese climate change

|  | Happiness | Satisfaction | Security | Tendency | Normalization | Capacity | Perseverance | Authenticity | Appropriateness | Reactivity | Constitutive | Value |
| --- | --- | --- | --- | --- | --- | --- | --- | --- | --- | --- | --- | --- |
| Beneficial ecological orientation | 39 | 65 | 60 | 33 | 37 | 212 | 197 | 67 | 941 | 89 | 67 | 1899 |
| Fuzzy ecological orientation | 0 | 2 | 2 | 2 | 2 | 5 | 4 | 3 | 17 | 1 | 0 | 41 |
| Destructive ecological orientation | 2 | 5 | 2 | 4 | 8 | 6 | 12 | 8 | 145 | 8 | 5 | 68 |
